# The Sanitary Condition and Vital Statistics of Fayette County, Kentucky

**Published:** 1851-12

**Authors:** W. S. Chipley

**Affiliations:** Lexington, Ky.


### WESTERN JOURNAL

OF

### MEDICINE AND SURGERY.

DECEMBER, 1851.

---

Art. I.—*The Sanitary Condition and Vital Statistics of Fayette County, Kentucky.* By W. S. Chipley, M. D., Lexington, Ky.

Fayette County, Ky., is situated in the central part of Northern Kentucky, washed on its South-East border by the Kentucky river, it is bounded at other points by the fertile counties of Jessamine, Woodford, Scott, Bourbon, and Clark. This part of the State was the home of the hardy and valiant pioneers who ventured to found settlements in the midst of dangers and far from the haunts of civilized man. The county was organised in 1781, having previously, with the counties of Jefferson and Lincoln, constituted the county of Kentucky.

The county is remarkable for the fertility, strength and durability of its soil, which is generally a heavy clay loam and mold. This deep stratum rests upon Silurian limestone, which is much used for building and fencing, and in the construction of the numerous turnpikes that now radiate from Lexington in almost every direction. There are also layers of a shaly or marly character.

Some of this limestone formation is exceedingly rich in fossil remains, and contains a large proportion of phosphate of lime, a portion of sulphate, and a small quantity of potash. Such is the productive nature of this soil, that our farmers have as yet paid but little attention to the means of improving and fertilizing their lands; but the material for enabling them to do so, abounds in many sections and will doubtless hereafter become important to the agriculturist.

At the period of its first settlement, this portion of Kentucky was famous for its majestic forests, abundant under growth and extensive cane-brakes. These have now almost entirely disappeared. Very little land in this county remains unenclosed; the under growth and cane-brakes have given way to a high state of cultivation and the dark impenetrable forest, have feeble representatives in the open wood-lands, clothed with a luxuriant growth of blue-grass. The prevailing timber is sugar-maple, white and black walnut, ash, black-locust and oak, honey-locust, hackberry, beach and hickory.

No valuable mines have been discovered in this county. Lead and zinc ores, with sulphate of barytes and fluor spar occasionally exist in the rock in small quantities.

Water for general use is obtained from springs and wells. It is hard, being impregnated with a portion of the bi-carbonates of lime and magnesia, and a small quantity of the phosphate of lime. It is believed that the use of this water favors the formation of urinary calculi in the human subject.

Prof. Gross in his recent able treatise on the urinary bladder thus refers to this opinion: "The opinion is plausible, and may be true, but how far, and to what extent, nobody has attempted to decide. If it be true that in Kentucky, Alabama, Tennessee, and Ohio, most calculous diseases occur in limestone regions, it is equally true that many are found in the freestone districts of those States."

Prof. Peter says, that the number of these cases has sensibly diminished in Lexington, since a more general resort has been had to rain water collected in cisterns. I have no doubt that the public health will improve as the custom of using rain water obtains more generally, especially in the city. My own observation leads me to believe, that the well water in the city is not as pure now as it was twenty-five or thirty years ago; this is certainly true of many wells in the more central parts of the city, several of them formerly noted for the excellency of the water they afforded, are now abandoned on account of their impurity. Neither springs nor streams abound in this county, and there is no marsh or swamp land. The cavernous nature of the sub-stratum on which the deep soil of this county rests renders sink-holes very common, and this formation, together with the elevated position of the country, may be the cause of the scarcity of water.—The farmers have very generally constructed artificial ponds to supply the wants of the numerous fine stock for which Kentucky is so justly celebrated. Seasons have occasionally occurred, when many farmers were compelled to drive their stock a considerable distance to water.

The city of Lexington, the county seat of Fayette, is in lat. 38° 2′ North, and long. 84° 26′, at an elevation of about eight hundred feet above the sea. It was established in 1779. The lower part of Elkhorn flows through Lexington, and is the common outlet for nearly the whole drainage of the city. The streets pass this stream by substantial brick arch-ways, and through the principal portion of the city. This rivulet is covered by large store-rooms, ware-houses, a rail-road depot, and market-houses. Professor Drake in his great work on "The Principal Diseases of the Valley of North America" says, "this stream as it flows off to the West, sinks into pools, in summer and autumn, and this creates a limited source of insalubrity, to the windward of the city." According to my recollection this statement answers to the condition of the country twenty or twenty-five years ago, but is no longer correct. The mills, with their mill-ponds of that period, have disappeared, and there is now seen but a small rivulet where, within my recollection, and this is in accordance with the recollection of old citizens with whom I have conversed, there flowed a bold stream, furnishing ample power to several mills that have now fallen into ruins, or wholly disappeared.

The geological character of this region induced one of the earliest travelers in the West to predict that, at some very distant day, a portion of Kentucky would become a waste—uninhabitable on account of a deficient supply of that absolute necessary of life, water. I do not believe that the failure will ever reach this extreme point; but I am sure that the volume of water in the Lower Fork of Elkhorn, as well as in many other small streams in this county, has sensibly diminished, and there are no longer "pools" or "spongy borders" to taint the atmosphere, or sicken those who reside in that vicinity.

The principal streets of Lexington are broad, running from South-East to North-West, being intersected by others at right-angles. They are nearly all well paved or macadamised with limestone, and are kept as clean as the streets of any city in the West. This important duty is faithfully performed by persons under the direction of the Mayor and City Council, and the expense is met by a moderate tax on the property of the city. The side walks are broad and well paved, and throughout the length of nearly every street these walks are bordered by shade trees of vigorous growth, affording protection from the intensity of the summer sun's rays, and imparting a rural and pleasing aspect to the whole city. The drainage is very perfect, and effected almost exclusively by surface drains. There are no pools, ponds or stagnant water within the city limits. The principal burial place was for many years on an elevated spot in the midst of the city, but no interments have been made there during the last eighteen years, it having been superceded by the Episcopal and Presbyterian cemeteries situated more distant from the mass of human habitations; and these are likely, in the course of a few years, to give place to the beautiful cemetery of forty acres, purchased two years ago by a number of spirited and liberal gentlemen, who have laid it out with excellent taste and spared no expense, to fit it for the holy purpose for which it is intended. This cemetery is at the extreme limit of the city, and distant from the habitations of the living.

The residences in the city are mostly brick, and there are few persons otherwise than comfortably lodged. It is rare to find more than one family occupying the same house. There are no under-ground tenants, and the poorest have the advantage of free ventilation. There are no closed courts, and but few human habitations opening on narrow confined alleys. The primitive log-cabin has, in the country, very generally given place to more aristocratic frame or brick buildings. The slave population is generally well lodged and clothed both in the city and country. All classes are abundantly fed with the most substantial fare, and even luxurious living is becoming far too common.

The county including the city is one of the wealthiest in the State, the real and personal estate being estimated at $17,861,843.

The great mass of the population is moral, intelligent, and religious. Churches are numerous and capable of accommodating in the aggregate 16,190 persons.

Much attention has always been given to education in this county. Transylvania Seminary was established in 1780. This institution, after its elevation to the dignity of a University, was, for many years, the leading literary institution of the West. A medical school, the finest in the West, was organized in 1817, and for many years ranked, in point of number of pupils, second only to the venerable institution at Philadelphia. A rapid decline led to a discontinuance of the winter, and the substitution of summer sessions. It is hoped that this venerable institution will flourish, under the new order of things, and soon re-attain her renown. Law and theological departments have flourished at different periods. The chairs in the law school are at this time filled by as able jurists and as competent and successful teachers as adorn any country.

Every neighborhood in the county is supplied with a good common school. Besides private male and female schools, the city has two spacious school houses, (and two more are about being provided) in which nearly one thousand children received the benefit of instruction from competent teachers during the past year. The city schools are open to every child, of suitable age, within the corporate limits free of expense. The first week of the present session has just elapsed, and six hundred and fifty children are already enrolled, and when, as is now contemplated, two other houses are provided, there will be ample facility for the education of twelve hundred children without expense to the parent. The city school houses were erected expressly for the purpose for which they are used, and are surrounded by ample play-grounds. Each is divided into a male and female department. A thorough English education is the aim of the city, but the languages are taught to those who desire to prepare to enter college. Each of the city schools is supplied with a well selected library, which has been the means of disseminating a great deal of information amongst parents as well as children.

The city and medical libraries contain each eight thousand volumes—the Law and University each, two thousand five hundred. There are fourteen private libraries in the county, containing in the aggregate twenty-eight thousand volumes. Almost every house boasts a good selection of volumes, and even amongst the poor books abound.

Serious offences are rare, and the jail is frequently almost or quite untenanted. On the first of June, 1850, there were only seven persons in prison, a majority of whom were slaves.

There is a county and a city poor house, but the means of subsistence are so abundant and so easily commended by moderate labor, that few, save a small number of old enfeebled persons, avail themselves of these generous provisions of the public. A physician appointed annually by the City Council, has charge of the city poor house, for which he receives moderate compensation.

The first Kentucky State Lunatic Asylum was established in Lexington. The enterprise was originally undertaken by a company of individuals, but was subsequently transferred to the State, and for nearly thirty years hundreds of unfortunates, with reason dethroned, have found here an Asylum, where every protection their cases demanded was humanely given, and every comfort they are capable of enjoying is afforded. I may as well say here, that the inmates of this institution are not enumerated in any of the tables that accompany this article.— They are a foreign population in our midst, sent to the Asylum from all parts of the State, with disordered intellects, the vital power of the whole system depressed, and the body in the most favorable condition to contract disease, and to suffer from any epidemic that may happen to invade the city. For these reasons we do not deem it proper to allude to this institution farther than to give the information contained in the following table, No. 1, exhibiting the number of inmates and the number of deaths, reported for the year ending June 1st, 1850:

TABLE No. 1.

| | *No. of Inmates.* | *No. of Deaths.* |
|---|---|---|
| Males,....... | 139 | 30 |
| Females,.... | 93 | 29 |
| Total,.... | 232 | Total, 59 |

The ravages of the cholera in 1833, left many friendless orphans too young to maintain themselves, and without a surviving relative to protect and support them. Of course in this wealthy and liberal community these helpless beings could not suffer from neglect. Several benevolent ladies, distinguished for their moral worth and deeds of charity, immediately raised a considerable sum of money, which they judiciously invested in a suitable lot of five acres, having on it a comfortable brick tenement, which they dedicated as an Orphan Asylum. The means for sustaining this noble charity were limited and precarious, hence its benefits were permitted to be enjoyed only by children who had lost both parents. Within the last two years however, this institution has received several bequests, and it now has in secure stocks, about ten thousand dollars, with the proceeds of which, and voluntary contributions, twelve or fifteen children are maintained in comfort and well educated. These children are under the charge of a matron by whom they are treated with parental tenderness; a school teacher resides in the house, and necessary servants are also provided. The directors of the institution, who are ladies, make regular and frequent visitations, and thus secure a faithful adherence to the judicious regulations which they have adopted for the government of the charity. Respectable young men, now actively engaged in useful employments, owe their maintenance and education to this benevolent institution, and some of its former inmates are excellent wives of some of Kentucky's best citizens. The children are well fed, neatly dressed, and always appear cheerful and happy. During nearly two years that I have given them professional services, no serious sickness has occurred amongst them, and not a single death has happened.—Fifty-five children have entered this institution since it was opened in 1833, of whom only three have died—one of croup, one of consumption, and one of measles. No more satisfactory evidence could be given of the careful and judicious management of the institution than this small bill of mortality. The children are of course from the most indigent families, and many of them have been unfortunately deprived of that training and regimen so necessary in early life, to impart vigor and solidity to the constitution, and yet only three out of fifty-five have perished, and one of that number of a deep-seated hereditary disease.

There is also in Lexington a "Female Benevolent Society" whose fair members have been ministering angels to to many deeply distressed and worthy persons who were suffering under the frowns of fortune. This society was organised in 1816, and not a year has since elapsed that it has not done much to alleviate human suffering—the needy widow, the sick and afflicted have been the peculiar objects of its bounty. Through this long period of time, the zeal of the members of this excellent society has suffered no abatement; with an ardor worthy of the impulses of their own benevolent hearts—without permanently invested funds, they have managed to secure contributions which have enabled them to dispense thousands, filling the lonely heart of the widow with joy, and soothing the last moments of many whose earthly hope had faded away. Many of the original founders of this society have passed from the scene of their charitable ministration to enter upon the rich reward of lives devoted to pure and unselfish benevolence—a few of their early associates yet remain, bright examples of those who have been uniformly actuated by;

"The noblest impulse generous minds can feel."

I take peculiar pleasure in giving this passing notice of a society which, within my own observation, has done so much to restore health to the sick, and afford comfort to the needy:

> "The drying up a single tear has more
> Of honest fame, than shedding seas of gore."

The prevailing diseases of Fayette, of a serious character, are typhoid fever and pneumonia. Within my own recollection remittent fever was the prevailing type, but I have met with but one well developed case of this fever since my return to Kentucky in 1844. Intermittent fever was formerly common on the small streams in different sections of the county, it is now exceedingly rare. An intelligent country practitioner informed me a few days since, that he had not observed ten cases during the last ten years.

Cholera has raged with terrible violence in this city and county at two different periods. This awful scourge of the human family carried off near five hundred persons in 1833, and over three hundred fell victims to it in 1849.—It prevailed in the Lunatic Asylum in 1849 about six weeks, beginning early in May, destroying fifty-nine of the inmates before it reached the city proper. Having disappeared from the Asylum, it began its ravages in the city on the 12th day of June, with fearful violence, and rapidly extended in every direction through the county. The mournful results are but too plainly exhibited in the following tables.

In August, 1850, this disease again appeared in the Asylum, and thirty persons fell victims to it, when it suddenly disappeared without spreading to the city, where there were but few cases of the disease, and not more than half a dozen deaths from that cause during the year.

Much speculation has been wasted in regard to the propogation of this disease, which has caused so many millions of the human family to perish. Some have asserted that the prevalence of limestone increases its fatality, and others regard it as a disease of malarious origin. No city in the world, of equal size, was ever freer from all the known sources of malaria than Lexington in 1849. I cannot speak of the condition of the city from personal observation in 1833, as I was then resident in the South.—But a few weeks before the disease appeared in the city in 1849, every citizen was actively engaged in having every source of malaria removed from cellars, back-yards and alleys, using that great purifier lime with a profusion that I never before witnessed. The streets were perfectly cleansed, the shade trees and even the gutters and curbstones white washed and lime in abundance was distributed wherever it was believed to be of the least use. The city authorities did all in their power to promote the cleansing process, furnishing lime to the citizens free of cost. Notwithstanding all this preparation, and a resort to these preventive measures, the disease appeared with a fatal violence that shrouded the whole county in mourning. I am sure that there were not less than three thousand cases, and over three hundred deaths, exclusive of those that occurred in the Asylum, marked the malignity of the epidemic. This disease presented here some of those singular features that have been remarked as characteristic of the malady elsewhere. Now it appeared as a wandering vagrant, having no fixed laws of progression or recession; and then its movements would correspond with astonishing uniformity with its course in former periods. Suddenly, without any apparent cause, it would invade a single street, and for a considerable space spare scarcely an individual, while an adjoining street not one hundred yards distant, would enjoy entire immunity. Expending its force here, in the course of twenty-four or forty-eight hours, it would again appear at some distant point, where perhaps the least apprehension was felt, and where no conceivable cause existed.

In 1833, after an apparent total cessation of the disease a gentleman of this city returned with his family to his residence. In a few days the disease appeared amongst his slaves, and nineteen fell victims, while every other part of the city was entirely free. Its course in 1849, was precisely similar, the same gentleman returned sometime after the epidemic was thought to be at an end; the disease revisited the house, and among others the proprietor and a daughter-in-law perished.

In 1833, Versailles, a village twelve miles distant, continued perfectly healthy, whilst death made almost every house in this city a place of mourning. Two years subsequently, matters were reversed, Versailles was sorely afflicted, and Lexington remained healthy.

In 1849, Versailles was exempt from the pestilence, and again suffered as before, two years after Lexington had passed the ordeal. Tne cause, progress and decline of the disease have been mysterious and inexplicable—no appreciable cause exists—no one can tell whence it comes or whither it goes. In 1849, observation taught us to anticipate increased mortality during the prevalence of easterly winds, but no one could tell in what part of the city the destructive scourge would fall.

Soon after the abatement of cholera in 1849, variola made its appearance, and in spite of the vigorous measures adopted by the authorities, spread rapidly through the city, and into the surrounding country. It raged as an epidemic. It had frequently visited the city before, but at no previous period was any difficulty experienced in checking its progress. Few cases occurred and death was rare. But in 1849, no vigilance could provide efficient checks to its spread. I do not know what number of persons suffered from this loathsome disease, but twenty-five deaths are reported in the census returns, and many others occurred after the first of June. It is thought that this disease is propagated only by contagion, but it is very certain that it is far more easily arrested in its course during some seasons than in others, indicating that, if propagated by contagion only, there are certain conditions of the atmosphere favorable to propagation. Such seemed to be the fact in this county in 1849, when more than ordinary care was taken to arrest the progress of the disease, but to no purpose.

The seasons are irregular and very variable. The range of temperature is great and vicissitudes extreme. The weather often continues quite agreeable until about Christmas, while the spring is frequently wet, cold and unpleasant. It is not uncommon for warm weather to set in early in the spring, and continue long enough to bring forward the fruit only to be destroyed by the frosts that follow. During the present year, after some weeks of mild pleasant weather, peaches and other fruits were entirely destroyed on the night of the first of May, having at that time attained considerable size. We rarely have much intense cold weather until after Christmas, on the setting in of which pneumonia becomes prevalent, and cases of this disease ordinarily continue to occur until April. July, August and September, are usually characterized by a high range of temperature, which however does not seem to be productive of any unusual sickness. One of the following tables will show November to be our most salubrious month, and this corresponds, very exactly, with my observation during several years past. It is notoriously a month of rest to the physician.

The following table exhibits a comparative view of the entire population of Fayette county, according to the census returns of 1840, and of 1850:

TABLE NO. 2.

| | *Whites.* | | *Free Col'd.* | | *Slaves.* | | |
|---|---|---|---|---|---|---|---|
| | Male. | Female. | Male. | Female. | Male. | Female. | Total. |
| 1850, - | 5609 | 5339 | 335 | 331 | 5447 | 5439 | 22500 |
| 1840, - | 5533 | 5352 | 298 | 301 | 5693 | 5017 | 22198 |
| Increase, | 76 | | 37 | 30 | | 422 | 565 |
| Decrease, | | 13 | | | 246 | | 259 |
| Total increase of population in ten years, | | | | | | | 306 |

While there appears to have been a small increase of population, including the city and county, there has really been considerable decrease in the population of the latter as distinguished from that of the former. According to the census of 1840, the population of the city of Lexington was 6,997, while according to the enumeration of the city assessors for 1850, (the city is not distinguished from the county in the census of 1850,) it numbered 7,890, showing an increase in the city alone of 893, an excess of 587 over the entire increase of the city and county when taken together.

The marriages for the year ending June 1st, 1850, were 119, or 1 in 92 of the entire white population.

In Massachusetts in 1849, the marriages were equal to 1 in every 143 inhabitants—the two extremes were in Hampden county, where there was 1 marriage to 105 inhabitants, and Berkshire, where there was only 1 to 335. This, according to the venerable Malthus, would indicate that the means of subsistence are more readily obtained here than in the Bay State, and the prospect of raising a family creditably more encouraging; hence the facility with which Western people contract marriage at an early age.

The following table No. 3, will show the number of marriages contracted in this county during each month, for five consecutive years, the sum total for each month for the whole term, etc.

For the convenience of comparison I have added the per centage of marriages during each month in Massachusetts for a term of six years, ending January 1st, 1850, during which there were 32,572 marriages.

TABLE NO. 3.

| | 1846. | 1847. | 1848. | 1849. | 1850. | Total. | Per Ct. | Mass. per ct. 6 years. |
|---|---|---|---|---|---|---|---|---|
| January, | 14 | 12 | 14 | 16 | 12 | 68 | 10.62 | 8.09 |
| February, | 8 | 9 | 11 | 11 | 11 | 50 | 7.81 | 5.72 |
| March, | 14 | 7 | 6 | 6 | 7 | 40 | 6.25 | 5.73 |
| April, | 8 | 5 | 6 | 7 | 5 | 31 | 4.84 | 8.83 |
| May, | 11 | 8 | 12 | 11 | 12 | 54 | 8.43 | 9.05 |
| June, | 6 | 10 | 7 | 7 | 11 | 41 | 6.40 | 7.75 |
| July, | 9 | 6 | 6 | 10 | 12 | 43 | 6.71 | 5.75 |
| August, | 6 | 10 | 10 | 8 | 10 | 44 | 6.87 | 6.10 |
| September, | 15 | 11 | 10 | 13 | 16 | 65 | 10.15 | 8.58 |
| October, | 17 | 14 | 15 | 10 | 19 | 75 | 11.71 | 10.58 |
| November, | 8 | 9 | 14 | 9 | 15 | 55 | 8.58 | 14.36 |
| December, | 11 | 12 | 17 | 15 | 19 | 74 | 11.56 | 9.46 |
| Total, | 127 | 113 | 128 | 123 | 149 | 640 | 99.93 | 100 |
| One in | 86.99 | 96.26 | 85 53 | 89. | 73.47 | | | |

I have no means of ascertaining the ages of the parties at the time these marriages were contracted, and cannot, therefore, give any information in regard to this interesting subject. We hope that the laudable efforts now being made to secure the passage of suitable registration laws will soon supply this defect, and afford many important and interesting views of social life.

In calculating the ratio of marriages for each year, white population alone is of course considered, as there is no record kept of the marriages of colored persons.—We think that Malthus announced as an invariable principle of population that the devastations of war, epidemics or famine were followed by an unusual number of marriages; this principle finds support in the above table, since the year following the great epidemic of 1849, gave 1 marriage to 73.47 of population, while the average of the four preceding years gave only 1 to 89.18.

The following table will show the number of each class who have survived the 70th year, and the proportion it bears to the whole number of each class.

### Table No. 4.

| *Whites.* | From 70 to 80 y'rs. | From 80 to 90. | From 90 to 100. | Over 100. |
|---|---|---|---|---|
| Male, | 75 or 1 in 74.78 | 16 or 1 in 350. | | |
| Female, | 66 " " " 80.90 | 20 " " " 266.95 | 3 or 1 in 1779.66 | 2 or 1 in 2669.05 |
| *Free col'd,* | | | | |
| Male, | 18 " " " 18.61 | 3 " " " 111.66 | 1 " " " 335.00 | |
| Female, | 22 " " " 15.04 | 6 " " " 55.16 | 2 " " " 165 50 | 1 " " " 331.00 |
| *Slaves.* | | | | |
| Male, | 35 " " " 155.61 | 11 " " " 495.18 | 2 " " " 2723.50 | |
| Female, | 43 " " " 126.48 | 13 " " " 417.61 | 11 " " " 494.45 | 4 " " " 1359.75 |
| Entire popul'n, | 259 " " " 86.87 | 69 " " " 326.98 | 19 " " " 1184.21 | 7 " " " 3214.28 |

From this table it appears that white males are more tenacious of life up to the 80th year, after which the expectation of life is in favor of the female, and that the probabilities continue to increase in her favor to the highest period of human existence. With slaves the probabilities are in favor of the female at the 70th year, and rapidly increase after that period.

In considering the small number of persons who have attained the age of 100 years, it should be recollected that the settlement of the county does not date 75 years, and that we are consequently indebted to emigration for all who exceed that time. As the number of these emigrants is small in proportion to the entire population, we cannot expect to find many persons living at the age of 100, and no inference can be legitimately drawn in regard to the climate as favorable or unfavorable to protracted life, from the small proportion which the extremely old bears to the whole population.

The large proportion of free colored persons who appear to attain old age is satisfactorily explained by the fact, that additions to this class of population are principally from the most temperate and faithful portion of our slaves, who have passed the dangers of childhood, and in many instances they have reached quite an advanced age before they are transfered from the slave to the free colored population. Almost all the old free colored persons have been slaves in the early part of their lives.

It is well enough also to be guarded against error in regard to the ages of slaves. While there is only 1 white female above 100 years old in 2669 persons, there appears to be 1 female slave of the same age to 1359. The age of a slave is rarely a matter of record, of course accuracy cannot be expected when the sole dependance is on the feeble memory of an ignorant domestic, or the uncertain calculations of the owner. With old slaves, age is held in great veneration, and after a certain period of life there is a strong disposition to magnify it in order to enjoy the consequence and dignity that are supposed to be its concomitants.

The following table No. 5, will show the average age of the living, the ratio of deaths, and the average age at death in the county of Fayette, including the city of Lexington, and also the ratio of deaths in the city proper.—Information from other places is added to furnish the means of comparison.

TABLE NO. 5.

| | Population. | Year. | Average age of the Living. | Year. | Ratio of Deaths. | Average Age at Death. |
|---|---|---|---|---|---|---|
| Boston, | 114,366 | 1845, | 23.5 | 1845, | 1 in 48.87 | 20.03 |
| New York, | 421,791 | 1846, | 23.1 | 1840, | " " 27.83 | 18. |
| Philadelphia, | 101,345 | 1845, | 24.1 | 1840, | " " 48.92 | 19.11 |
| | | | *Whites.* | | | |
| Baltimore, | 124,331 | 1848, | 23.1 | 1840, | " ". 29.37 | 19.09 |
| United States, | | | 22.2 | | | |
| Lexington, Ky. | 7,959 | 1850, | | 1850, | " " 53.41 | |
| | | | *Whites & Free col'd.* | | | |
| Fayette Co. | 22,500 | '49-'50 | 23.65 | '49-'50 | " " 30.28 | 26.09 |

The average age of the living slaves is less than twenty years. This diminished average is doubtless owing in part at least to the fact that the exportation of this class of persons to the South is almost limited to adults, leaving all the children to reduce the average age of those who remain. The exportation is mostly of able bodied males, and this trade has greatly increased within the last ten years, insomuch that it has absorbed 246 persons in addition to the natural increase.

Excluding the Lunatic Asylum, as I have done, there were 743 deaths. The following table No. 6, will show the causes of death arranged alphabetically, the number of each class of persons who died of different diseases, the number who perished of each disease at certain ages, and the total number of deaths from each cause, as reported in the census returns.

TABLE No. 6.

| *Cause of Death.* | White Male, | White Female, | Free col'd Male, | Fr. col'd Female, | Male Slaves, | Female Slaves, | Under one year, | One and under 5 | 5 to 10, | 10 to 20, | 20 to 30, | 30 to 40, | 40 to 50, | 50 to 60, | 60 to 70, | 70 to 80, | 80 to 90, | 90 to 100, | 110, | Total, |
|---|---|---|---|---|---|---|---|---|---|---|---|---|---|---|---|---|---|---|---|---|
| Apoplexy, | 3 | 2 | | | 1 | | | | | | | 3 | 1 | | 1 | 1 | | | | 6 |
| Accident, | 6 | | | | 8 | 6 | 6 | 3 | 6 | 1 | | 1 | 1 | 2 | | | | | | 20 |
| Bowels Inflam. of | 1 | | | | | | | | | | | | | | 1 | | | | | 1 |
| Bronchitis, | 1 | 1 | | | 1 | | | | | | 2 | | | | 1 | | | | | 3 |
| Brain Dis. of | 4 | 3 | 1 | | | | | 6 | | | | | | | 2 | | | | | 8 |
| Cholera, | 88 | 59 | 14 | 8 | 86 | 55 | 19 | 29 | 19 | 42 | 76 | 41 | 36 | 18 | 17 | 7 | 3 | 3 | | 310 |
| Consumption, | 3 | 1 | | 1 | 10 | 9 | | 3 | 3 | 9 | 4 | 1 | 3 | | 1 | | | | | 24 |
| Cancer, | 1 | 1 | | | | 1 | | | | | | 1 | | 1 | | 1 | | | | 3 |
| Cholera Morbus, | 1 | | | | | | | 1 | | | | | | | | | | | | 1 |
| Cold, | 1 | | | 1 | 2 | 1 | 4 | 1 | | | | | | | | | | | | 5 |
| Child Bed, | | | | | | 1 | | | | 1 | | | | | | | | | | 1 |
| Cholera Infantum, | 3 | | | | | 1 | | 4 | | | | | | | | | | | | 4 |
| Croup, | | | 1 | | 3 | 2 | 5 | 1 | | | | | | | | | | | | 6 |
| Chronic, | 32 | 20 | 1 | 4 | 30 | 24 | 10 | 13 | 7 | 11 | 13 | 18 | 10 | 9 | 6 | 7 | 6 | | 1 | 111 |
| Chills and Fever, | | | | | 1 | | | | | | | | 1 | | | | | | | 1 |
| Dropsy, | 2 | 2 | | | 3 | 3 | 1 | | | 1 | 1 | 1 | | 1 | 4 | 1 | | | | 10 |
| Diarrhæa, | 1 | 2 | | | | | | | 1 | | 1 | | | | | 1 | | | | 3 |
| Dyspepsia, | 1 | | | | | | | | | | 1 | | | | | | | | | 1 |
| Dysentery, | 2 | 6 | | | 3 | 1 | 1 | 3 | 2 | 2 | 1 | 2 | | | 1 | | | | | 12 |
| Epilepsy, | 2 | | | | | | | | | | | 1 | 1 | | | | | | | 2 |
| Fever, | 4 | 2 | | | 6 | 7 | 3 | 5 | 1 | 3 | 6 | | | | 1 | | | | | 19 |
| Fever, Typhoid | 10 | 10 | 2 | | 12 | 14 | 1 | 1 | 3 | 18 | 16 | 4 | 5 | | | | | | | 48 |
| Fever, Scarlet | 2 | 3 | | | | 1 | | 6 | | | | | | | | | | | | 6 |
| Gravel, | 1 | | | | | | | | | | | | | | 1 | | | | | 1 |
| Heart, Dis. of | 1 | 1 | | | | 3 | 1 | | | | 2 | 1 | | | 1 | | | | | 5 |
| Intus-susception, | | | | | 1 | | | | | | 1 | | | | | | | | | 1 |
| Lock-Jaw, | | | | | | 1 | | | 1 | | | | | | | | | | | 1 |
| Lungs, Conges. of | | | | | | 1 | | 1 | | | | | | | | | | | | 1 |
| Murdered, | 1 | | | | | | | | | | | | 1 | | | | | | | 1 |
| Old Age, | 1 | | | 1 | | 3 | | | | | | | | | | 2 | 2 | 1 | | 5 |
| Pleurisy, | | | | | 1 | | | | | | 1 | | | | | | | | | 1 |
| Peritonitis, | 1 | | | | | | | | | 1 | | | | | | | | | | 1 |
| Pneumonia, | 4 | 4 | | | 6 | 6 | 4 | 5 | | | 2 | 3 | 4 | 1 | 1 | | | | | 20 |
| Small Pox, | 10 | 2 | 5 | | 6 | 2 | 1 | 2 | 1 | 3 | 6 | 4 | 3 | 4 | 1 | | | | | 25 |
| Scrofula, | | | | 1 | 3 | 3 | 1 | 1 | 1 | 2 | 1 | | | | 1 | | | | | 7 |
| Spasms, | 1 | 2 | | | 1 | 1 | 1 | 2 | 1 | | | | | 1 | | | | | | 5 |
| Sudden, | 3 | 7 | | | 14 | 5 | 12 | 7 | 1 | | 6 | 1 | 1 | 1 | | | | | | 29 |
| Unknown, | 3 | 5 | | | 13 | 8 | 14 | 3 | 3 | 1 | 5 | 1 | 1 | 1 | | | | | | 29 |
| Worms, | | | | | 2 | 4 | | 2 | 4 | | | | | | | | | | | 6 |
| Total, | 194 | 133 | 24 | 16 | 213 | 163 | 84 | 99 | 54 | 95 | 145 | 83 | 68 | 39 | 40 | 20 | 11 | 4 | 1 | *743 |

Table No 7, will present in compact form the number of deaths in each month from cholera, fever, typhoid fever, pneumonia, and other diseases respectively; also the per centage of deaths for each month excluding the cholera.

*Three persons are enumerated in the census tables twice, and there was one still-born not included in this table.

TABLE No. 7.

| | Cholera, | Fever, | Typhoid Fever, | Pneumonia, | Other Diseases, | Total, | Per centage of Deaths exclu-ding Cholera. |
|---|---|---|---|---|---|---|---|
| January, | | 1 | 5 | 2 | 46 | 54 | 12.47 |
| February, | | 1 | 3 | 2 | 33 | 39 | 9. |
| March, | 1 | | 4 | 6 | 34 | 45 | 10.96 |
| April, | | | 2 | 2 | 25 | 29 | 6.69 |
| May, | 1 | 1 | 6 | 3 | 29 | 40 | 9. |
| June, | 36 | 3 | 5 | 3 | 35 | 82 | 10.62 |
| July, | 225 | 3 | 5 | | 30 | 263 | 8.77 |
| August, | 35 | 6 | 6 | | 26 | 73 | 8.77 |
| September, | 7 | 2 | 3 | | 21 | 33 | 6. |
| October, | 5 | | 4 | | 21 | 30 | 5.77 |
| November, | | | | 1 | 13 | 14 | 3.23 |
| December, | | 2 | 5 | 1 | 33 | 41 | 9.46 |
| Total, | 310 | 19 | 48 | 20 | 346 | 743 | 99.94 |

November thus appears to be our healthiest month, and this is sustained by several years' observation. This table also shows the prevalence of typhoid fever throughout the year; indeed, according to my observation, this fever is peculiar to no season, and has not seemed to be modified by winter or summer, spring or autumn.

Of those who died 79 were natives of Virginia; 12 of Maryland; 10 of England; 7 of Ireland; 7 of New York; 5 of Pennsylvania; 4 of Tennessee; 3 each of New Jersey, North Carolina and Delaware; 2 each of South Carolina, Arkansas, Missouri, and Scotland; 1 each of Connecticut, Massachusetts, Indiana, Mississippi, and Germany; 3 unknown; and the remainder 594 of Kentucky.

The returns afford very little satisfaction in regard to the occupations of the deceased; there were 36 farmers whose aggregate ages amounted to 1656 years, giving an average of 45.97; while the aggregate ages of 45 mechanics amounted to only 1569 years, an average of 34.86, showing as far as this limited number goes, the expectation of life in favor of the farmer by 11.11 years. This fact should awaken the attention of every philanthropic legislator, and if any thing can be done to ameliorate the condition and protract the life of the working men in our cities, the simple dictates of humanity demand that it should be no longer neglected. We want more information on this subject, and its many important kindred subjects, and this can be had only by a law for the uniform registration of births, marriages and deaths. Such a law has been in force in the enlightened State of Massachusetts for seven years, and so obvious are the benefits resulting from it, that nothing could induce the people to dispense with it. From the last report, 1850, we learn that 4,974 farmers died at an average age of 63.83 years; 2,283 laborers averaged only 45.39 years; 662carpenters averaged 49.28 years; 1,011 shoe-makers averaged 43.04 years. This immense difference in the probabilities of life, among persons engaged in different pursuits, will naturally attract attention, and it is hoped that many of the evils that press heavily upon the poor may be mitigated. The effect of occupation does not seem to have received the consideration it merits from Life Assurance Companies; the farmer is charged the same premium as those who cannot reasonably expect to live as long by 10 or 20 years.

Table No. 8, will show the population classified; the number of deaths in each class, the ratio of deaths, and the per centage of mortality.

TABLE NO. 8.

| | Population. | Deaths. | One in | Per Centage. |
|---|---|---|---|---|
| White Males, | 5,609 | 194 | 28.91 | 3.45 |
| White Females, | 5,339 | 133 | 40.14 | 2.49 |
| White Population, | 10,948 | 327 | 33.48 | 2.98 |
| Free Colored Males, | 335 | 24 | 13 95 | 7.16 |
| Free Colored Females, | 331 | 16 | 20.68 | 4.83 |
| Free Colored Population, | 666 | 40 | 16.65 | 6. |
| Slave Males, | 5,447 | 213 | 25.57 | 3.91 |
| Slave Females, | 5,439 | 163 | 33 36 | 2.99 |
| Slave Population, | 10,886 | 376 | 28.95 | 3.45 |
| Entire Population, | 22,500 | 743 | 30.28 | 3.30 |

The preceding table exhibits the absolute mortality during the year, but it does not represent fairly the ordinary mortality in this county. The returns, from which the table is made up, embrace the period of the prevalence of a most malignant epidemic cholera, a disease which has made its appearance amongst us but once since 1833, and which we fondly hope we may never again encounter. Excluding the deaths from this cause will enable us to approximate the ordinary mortality of the county; I have therefore, constructed the following table No. 9, with this exclusion.

TABLE NO. 9.

| | Population. | Deaths Exclusive of Cholera. | One in | Per Cent. |
|---|---|---|---|---|
| White Males, | 5,609 | 106 | 52.91 | 1.88 |
| White Females, | 5,339 | 74 | 72.14 | 1.39 |
| White Population, | 10,948 | 180 | 60.82 | 1.64 |
| Free Colored Males, | 335 | 10 | 33.50 | 2.98 |
| Free Colored Females, | 331 | 8 | 41.37 | 2 41 |
| Fre Colored Population, | 666 | 18 | 37. | 2.70 |
| Slave Males, | 5,447 | 127 | 42.88 | 2.34 |
| Slave Females, | 5,439 | 108 | 50.36 | 1.98 |
| Slave Population, | 10,886 | 235 | 46.32 | 2.15 |
| Entire Population, | 22,500 | 433 | 51.96 | 1.92 |

By a glance at table No. 6, it will be perceived that 111 deaths are ascribed to chronic diseases without any more definite information in regard to the character of the malady of which those persons perished, and it is remarkable that of this large number, 110 are reported by one of the two gentlemen who received the census returns in this county. But in this half of the county, where this uncertain note of the cause of death occurs so frequently, not a single death is ascribed to consumption, disease of the heart, scrofula, dropsy, or old age; all of which are probably included under this indefinite term chronic. But we may approximate the number that probably died of these several diseases.

The entire population of district No. 2, is returned at 9,818, and gives 24 deaths of consumption. The population of district No. 1, is 12,682, and if, as is probable, deaths by consnmption bear the same relation to population in this as in the other half of the county, about 31 died of this disease, and should be subtracted from the chronic and added to the 24 consumption, reported in No. 2, making in all 55.

By the same mode of calculating the probable number of deaths from disease of the heart, we shall have 11 instead of 5;—of scrofula 16 instead of 7; of dropsy 22 instead of 10; of old age 11 instead of 5. This calculation abstracts 70 from the list of chronic diseases, and leaves but 41 to be accounted for.

Disease of the brain includes, Inflammation 3, congestion 1; brain fever 2; brain disease 2.

Accident includes, Smothered 6; wounded 3; fall, burnt, scalded, each 1.

Twenty-five deaths from small pox are reported, a larger number probably than have occurred in this county, from the same cause, during fifty years prior to 1849. Occasionally this loathsome disease has been imported into the city, but heretofore, we have found no difficulty in arresting its progress and limiting it to the few individuals who had been unfortunately exposed to the contagion before it was discovered to be in our midst. But in 1849–'50, as already stated, it seemed to break over all restraints, and spread rapidly through town and country.

Of this disease one died on the 3d day; three on the 6th; one on the 7th; two on the 9th; eleven on the 10th; three on the 11th; one on the 14th; two on the 15th; and one on the 17th. Average duration of these cases precisely ten days.

Of pneumonia, three died on the 4th day; one on the 6th; two on the 9th; nine on the 10th; one on the 12th; one on the 15th; one on the 20th; one on the 42d; and one unknown. Average duration of these cases a fraction over eleven days.

Table No. 10, will show the duration of fever, and typhoid fever, with the different classes of persons who perished of these forms of disease.

TABLE No. 10.

| | Typhoid Fever: *Number of Days Sick.* | | | | | | | Fever: *Number of Days Sick.* | | | | | | |
|---|---|---|---|---|---|---|---|---|---|---|---|---|---|---|
| Days. | White Males. | White Females. | Free Black Males. | Free Black Females. | Male Slaves. | Female Slaves. | Total Number. | White Males. | White Females.* | Free Black Males. | Free Bluck Females. | Male Slaves. | Female Slaves. | Total. |
| 4 | | | | | | | | | | | | 1 | 1 | 2 |
| 5 | | | | | | | | | | | | | 1 | 1 |
| 6 | 1 | 1 | | | 1 | 1 | 4 | | | | | | | |
| 7 | 1 | | | | | 2 | 3 | | | | | | | |
| 8 | | | | | | 1 | 1 | | | | | | | |
| 9 | | 1 | | | | 1 | 2 | | | | | | | |
| 10 | | 1 | | | 4 | 2 | 7 | | | | | | | |
| 12 | | | | | | 2 | 2 | 1 | | | | | | 1 |
| 13 | | | | | 1 | | 1 | | | | | 1 | | 1 |
| 14 | 1 | | | | 1 | 2 | 4 | 1 | | | | | 2 | 3 |
| 15 | | 1 | | | | | 1 | | | | | | | |
| 16 | | 1 | | | | | 1 | | | | | | | |
| 18 | | | | | | | | | 1 | | | | | 1 |
| 20 | 1 | 3 | | | | | 4 | | | | | 1 | | 1 |
| 21 | 2 | 1 | 2 | | 1 | 1 | 7 | | | | | | | |
| 22 | 1 | | | | | | 1 | | | | | | | |
| 28 | 1 | | | | 1 | | 2 | | | | | | | |
| 30 | 1 | | | | | 1 | 2 | 1 | | | | | 1 | 2 |
| 35 | | | | | | | | 1 | | | | | 1 | 2 |
| 40 | | | | | 1 | | 1 | | | | | | | |
| 45 | 1 | | | | | | 1 | | | | | | | |
| 49 | | | | | | 1 | 1 | | | | | | | |
| 50 | | | | | 1 | | 1 | | | | | | | |
| 60 | | | | | 1 | | 1 | | | | | 3 | 1 | 4 |
| 90 | | 1 | | | | | 1 | | | | | | | |
| | 10 | 10 | 2 | 00 | 12 | 14 | 48 | 4 | 1 | 00 | 00 | 6 | 7 | 18 |

*One case noted as chronic—time sick not given.

Table No. 11, will show the average number of days that individuals of each class of persons were sick of fever, and of typhoid fever.

TABLE No. 11.

| | White Male. | White Female. | Free Colored Male. | Free Colored Female. | Male Slaves. | Female Slaves. | Total Average. |
|---|---|---|---|---|---|---|---|
| Typhoid Fever, | 21.04 | 22.7 | 21. | | 22.6 | 14.9 | 20.08 |
| Fever,.......... | 22.75 | 18. | | | 36.16 | 23.14 | 26.61 |

Throwing the two forms of fever together, the average duration of the cases embraced in the table is 21.86 days.

Forty-eight deaths are reported as resulting from typhoid fever, and nineteen from fever. An inspection of the preceding tables will satisfy any one, that most of the latter ought to have been reported with the former. The prevailing form of fever in this county is typhoid, and I have not witnessed a death from any other type of fever during the last seven years that I have resided in Lexington. During twelve years residence in Georgia, I was familiar with remittent and intermittent fevers, but these types are rare in this county—I do not recollect to have met with but one case of the remittent fever.

By reference to table No. 6, it will be perceived that of those who died of typhoid fever, one was less than one year old; one between one and five; three between five and ten; eighteen between ten and twenty; sixteen between twenty and thirty; four between thirty and forty; and five between forty and fifty.

The number of each class who died of cholera, at certain ages, during the year ending June 1st, 1850, will be perceived by a glance at table No. 12. Table No 13, is similarly constructed in regard to other diseases.

TABLE NO. 12.

| | Under 1 year. | 1 to 10. | 10 to 20. | 20 to 30. | 30 to 40. | 40 to 50. | 50 to 60. | 60 to 70. | 70 to 80. | 80 to 90. | 90 to 100. | Total. |
|---|---|---|---|---|---|---|---|---|---|---|---|---|
| White Males, ............. | 9 | 15 | 12 | 19 | 13 | 8 | 5 | 3 | 3 | | 1 | 88 |
| White Females, ............. | 4 | 9 | 11 | 8 | 5 | 7 | 5 | 5 | 2 | 2 | 1 | 59 |
| Free Colored Males, ......... | | | 2 | 3 | | 3 | 2 | 1 | 1 | 1 | 1 | 14 |
| Free Colored Females, ....... | | 1 | 2 | 2 | 1 | | | 1 | 1 | | | 8 |
| Male Slaves, ............... | 5 | 13 | 4 | 29 | 19 | 10 | 4 | 2 | | | | 86 |
| Female Slaves, ............. | 1 | 10 | 11 | 15 | 3 | 8 | 2 | 5 | | | | 55 |
| | 19 | 48 | 42 | 76 | 41 | 36 | 18 | 17 | 7 | 3 | 3 | 310 |

Of other diseases:

TABLE NO. 13.

| | Under 1 year. | 1 to 10. | 10 to 20. | 20 to 30. | 30 to 40. | 40 to 50. | 50 to 60. | 60 to 70. | 70 to 80. | 80 to 90. | 90 to 100. | 110. | Total. |
|---|---|---|---|---|---|---|---|---|---|---|---|---|---|
| White Males, .......... | 9 | 20 | 6 | 21 | 17 | 10 | 5 | 9 | 5 | 4 | | | 106 |
| White Females, ......... | 8 | 13 | 12 | 19 | 8 | 3 | 2 | 5 | 3 | 1 | | | 74 |
| Free Colored Males, ..... | | 2 | 2 | 4 | 2 | | | | | | | | 10 |
| Free Colored Females, .. | 1 | 2 | 1 | | 2 | | | 1 | 1 | | | | 8 |
| Male Slaves, .......... | 19 | 36 | 14 | 16 | 8 | 12 | 12 | 6 | 2 | 1 | 1 | | 127 |
| Female Slaves, ........ | 28 | 32 | 18 | 9 | 5 | 7 | 2 | 2 | 2 | 2 | | 1 | 108 |
| | 65 | 105 | 53 | 69 | 42 | 32 | 21 | 23 | 13 | 8 | 1 | 1 | 433 |

It may not be uninstructive to introduce here a meteorological table for 1849, although it is necessarily imperfect. The table was registered by Dr. R. P. Hunt and the writer, and in explanation of the manner of noting the terms used in connection with rain, it is only necessary to say that we had no water guage.

The observations, registered here, were made three times a day; at 7, A. M., and at 3, and 10, P. M. Our Barometer was not a perfect instrument, and the range given may not, in all cases, be perfectly accurate. These explanations are sufficient for an apology for the deficiencies of the abstract.

*Abstract of a Meteorological Journal for* 1849, *from April* 1*st to October* 21*st inclusive; at Lexington, Ky., Latitude* 38° 2′ *North, Longitude* 84° 26′ *West.*

| Weekly. | Therm. Max. | Therm. Min. | Therm. Range. | Barometer. Max. | Barometer. Min. | Barometer. Range. | Remarks. |
|---|---|---|---|---|---|---|---|
| April 1st,..... | 70 | 46 | 24 | 29 3 | 28 6 | 0.7 | Rain on the 4th. |
| " 8th,..... | 74 | 44 | 30 | 29.4 | 28.5 | 0 9 | Rain on the 8th and 9th. |
| " 15th,.... | 58 | 34 | 24 | 29.2 | 28.6 | 0.6 | Rain 17th; snow 18th; heavy frost 20th; slight sprinkle 21st. |
| " 22d, .... | 74 | 51 | 23 | 29.1 | 28.6 | 0 5 | Rain 23d; shower 27th. |
| " 29th,.... | 80 | 58 | 22 | 29.2 | 28.3 | 0 9 | Rain 30th; 3d and 5th. |
| May 6th,.... | 76 | 54 | 22 | 28.9 | 28.4 | 0.5 | Rain 6th and 12th. |
| " 13th,.... | 70 | 54 | 16 | 28.9 | 28.6 | 0.3 | Sprinkle 15th; rain 16th;—*Cholera, 5 cases 1 death 19th.* |
| " 20th,.... | 76 | 56 | 20 | 29. | 28 5 | 0.5 | |
| " 27th,.... | 78 | 59 | 19 | 29. | 28.4 | 0.6 | Rain 29th and 2d. |
| June 3d, .... | 84 | 68 | 16 | 28.7 | 28 3 | 0.4 | Rain 6th and 8th; copious rain, thunder and lighting 9th. |
| " 10th,.... | 81 | 64 | 17 | 28.9 | 28 5 | 0.4 | Rain 11th, 13th, 14th and 15th. |
| " 17th,.... | 89 | 68 | 21 | 29. | 28.3 | 0.6 | Slight rain 23d. |
| " 24th,.... | 89 | 72 | 17 | 28.6 | 28.2 | 0.4 | Rain 24th, 25th, 27th, 29th and 30th. |
| July 1st,.... | 77 | 64 | 13 | 28.9 | 28.5 | 0.4 | Rain 1st and 8th; much rain the 7th. |
| " 8th,.... | 86 | 72 | 14 | 28.7 | 28.4 | 0.3 | Much rain 9th. |
| " 15th,.... | 80 | 68 | 12 | 28.9 | 28.4 | 0.5 | Much rain 19th. |
| " 22d,..... | 80 | 70 | 10 | 28.8 | 28.3 | 0.5 | Much rain 24th, 25th and 27th. |
| " 29th,..... | 82 | 62 | 20 | 29. | 28.4 | 0.6 | Much rain 29th and 30th; rain on the 4th. |
| Aug. 5th,..... | 84 | 66 | 18 | 28.8 | 28 3 | 0.5 | Rain 8th. |
| " 12th,..... | 82 | 67 | 15 | 28.9 | 28.5 | 0.4 | Rain 13th, 14th, and 17th. |
| " 19th,..... | 86 | 70 | 16 | 28.7 | 28.3 | 0.4 | Rain 20th. |
| " 26th,..... | 82 | 69 | 13 | 29. | 28.4 | 0.6 | Rain 28th, 29th. |
| Sept. 2d,..... | 80 | 60 | 20 | 29.1 | 28.5 | 0 6 | Rain 4th. |
| " 9th,..... | 84 | 60 | 24 | 29.2 | 28.4 | 0.8 | |
| " 16th,..... | 80 | 67 | 13 | 28.9 | 28.4 | 0.5 | Rain 28th. |
| " 23d, .... | 78 | 56 | 22 | 29. | 28.5 | 0.5 | |
| " 30th,..... | 72 | 50 | 22 | 29. | 28.3 | 0.7 | Rain 30th, 2d, and 6th. |
| Oct. 7th,..... | 56 | 50 | 6 | 29.1 | 28 7 | 0.4 | |
| " 14th,.... | 68 | 48 | 20 | 29.3 | 28.5 | 0.8 | Rain 15th and 16th. |
| " 21st,..... | 64 | .. | .. | 28.7 | .... | ... | Rain. |

At this time the barometer was accidentally injured, and the Journal was discontinued.

To ascertain the relative mortality at different epochs of life, it is necessary to introduce another element; we must know the number of the living at these different epochs. I have ascertained this by a calculation based on the census returns of 1840, when the entire population was only three hundred and six less than in 1850.

The following table gives the results:

TABLE NO. 14.

| | Male Population, | Deaths from Cholera; per cent... | Deaths from Other Diseases; per cent....... | Female Population,....... | Deaths from Cholera; per cent... | Deaths from Other Diseases; per cent....... | Population Total, | Deaths from all Causes; per ct.. |
|---|---|---|---|---|---|---|---|---|
| Under 10 years,... | 3178 | 1.32 | 2.70 | 3050 | .81 | 2.75 | 6228 | 3.80 |
| From 10 to 20, .... | 2676 | .67 | .82 | 2928 | .81 | 1.05 | 5604 | 1.69 |
| " 20 " 30, .... | 2324 | 2.19 | 1.76 | 1978 | 1.25 | 1.41 | 4302 | 3.37 |
| " 30 " 40, .... | 1340 | 2.38 | 2.01 | 1251 | .71 | 1.19 | 2591 | 3.20 |
| " 40 " 50, .... | 860 | 2.44 | 2 55 | 838 | 1.79 | 1.19 | 1698 | 4.00 |
| " 50 " 60, .... | 508 | 2.16 | 3.34 | 577 | 1.21 | .69 | 1085 | 3.69 |
| " 60 " 70, .... | 321 | 1.87 | 4.67 | 344 | 3 19 | 2.32 | 665 | 6.01 |
| " 70 " 80, .... | 128 | 3.12 | 5.46 | 131 | 2.29 | 4.58 | 259 | 7.72 |
| " 80 " 90, .... | 30 | 3.33 | 16.66 | 39 | 5.13 | 7.69 | 69 | 15.94 |
| " 90 " 100, ... | 3 | 66.66 | 33.33 | 16 | 6.25 | | 19 | 21.05 |
| Over .... 100, ... | | | | 7 | | 14.28 | 7 | 14.28 |

The above proportions of population divided into decades, are ascertained by calculation to the seventieth year, the remainder is by actual enumeration. In looking over the aggregate, I find that I have an excess of twenty-seven persons, but distributed as it is, it can make no material difference, and I have not therefore sought to correct it.

From this table it would appear, that males from 10 to 20 years old, were less subject to cholera than during any other decade of life—then follows with increasing liability the period intervening between birth and 10 years of age—then from 60 to 70—50 to 60—20 to 30—30 to 40—40 to 50; after 70, the table shows an increasing liability with advancing age, but the number of persons submitted to observation is too small to settle a principle.

Following the same order with the male population, in regard to diseases other than cholera, we find the least mortality between 10 and 20, then between 20 and 30;—30 and 40;—40 and 50;—under 10; after 50, the mortality increases with age.

With females, the least mortality from cholera was from 30 to 40;—exactly equal under 10, and from 10 to 20; then follows from 50 to 60; from 20 to 30;—from 40 to 50;—from 70 to 80;—from 60 to 70; etc.

From other diseases, females suffered less from 50 to 60;—then from 10 to 20;—30 to 50;—20 to 30;—60 to 70; —under 10; etc.

In the aggregate and from all diseases, the least mortality is found between the ages of 10 and 20;—the mortality of the different epochs increasing in the following order; from 30 to 40;—20 to 30;—50 to 60; under 10;—40 to 50, etc.

According to observations in Lowell, from 1841 to 1845, the probabilities of surviving the present period of life were the greatest between the ages of 20 and 30; the next greatest between 10 and 20; then followed between 30 and 40;—40 and 50;—50 and 60;—under 10; and the least over 60.

In Boston, the results were similar, but commencing as with us, one decade earlier in life.

This table confirms the opinion commonly entertained, of the healthfulness of this climate for children, and the opinion is still further strengthened by table No. 6, where few of those diseases appear which constitute the great outlets of infantile life.

But, there is still more positive proof of this, when we compare the mortality of persons under 10 years of age in this county, with the results obtained under the registration laws of Massachusetts. The report of 1850, shows that of all who die in the cities of that State, 49.81 per cent. are under 10 years of age; in the country, the same decade of life furnished 41.11 per cent of the whole number of deaths; in the whole State, the loss at this age was 45.11.

In the county of Fayette during the year, ending June 1st, 1850, the whole number of deaths was 743, of which only 31.89 per cent. were under 10 years of age.

In Lowell, statistics showed that the expectation of life between the ages of 10 and 30, was seven times greater than it was with those under 10 years old.

In Fayette county, of those under 10 years old, 1 died in 26.28; of those between 10 and 30, 1 died in 41.27; so that with us the expectation of life between 10 and 30, is less than twice as great as with those under 10 years of age.

To show how injuriously we are affected by calculations based upon returns that include the dreadful epidemic cholera of 1849, we present the following statistics from the city assessors' books, for the year ending Jan. 10th, 1850. There were at that time:

| | |
|---|---|
| White Males, - - - - - - | 2,578 |
| White Females, - - - - - | 2,534 |
| Colored Males, - - - - - | 1,278 |
| Colored Females, - - - - | 1,510 |
| Total, - - - - - - - | 7,890 |

Excluding the Lunatic Asylum, the deaths were 406, or 1 in 19.43, or 5.65 per cent.—an unusual and terrible mortality.

Now, compare with this the returns of the following year, ending Jan. 10th, 1851, during which there was an ordinary amount of sickness, and the usual number of deaths. There were then enumerated:

| | |
|---|---|
| White Males, - - - - - - | 2,615 |
| White Females, - - - - - | 2,604 |
| Colored Males, - - - - - - | 1,183 |
| Colored Females, - - - - | 1,557 |
| Total, - - - - - - - - | 7,959 |

The deaths were 149, or 1 in 53.41, or 1.89 per cent.—I am satisfied that the average mortality of Lexington for a series of years, would not exceed this.

The births in Lexington in 1850, were to the population as 1 to 33 nearly. In the enumeration of births, there is no distinction of sex or color; the only fact ascertained is the absolute number born within the year. It is hoped that a better system will be adopted next year, and fuller information obtained in regard to the sanitary condition and vital statistics of the city. If this is done, I have no doubt that Lexington will be found to be as salubrious as any city of the same population in this country.

In closing these vital statistics, it may be a matter of some interest to refer to the medical corps of Fayette county. The most reliable information in my reach, shows that there are in this county, forty-four regular physicians, two homœopathists, and two botanics, making in all forty-eight, or one to every four hundred and sixty-nine inhabitants.

